# The impact of polygenic risks on neurotransmitter‐related grey matter atrophy in Alzheimer’s disease

**DOI:** 10.1002/alz.088738

**Published:** 2025-01-09

**Authors:** Riccardo Manca, Micaela Mitolo, Maria Giulia Bacalini, Sabina Capellari, Annalena Venneri

**Affiliations:** ^1^ Brunel University London, Uxbridge UK; ^2^ University of Parma, Parma Italy; ^3^ IRCCS Istituto delle Scienze Neurologiche di Bologna, Bologna, Italy Italy; ^4^ IRCCS Istituto delle Scienze Neurologiche di Bologna, Bologna Italy; ^5^ University of Bologna, Bologna Italy; ^6^ Brunel University London, London UK

## Abstract

**Background:**

Tau protein tangles have been recently shown to accumulate in multiple brainstem nuclei in pre‐cortical Alzheimer’s disease (AD) stages. The impact of neurotransmission alterations on brain atrophy and their genetic correlates in AD remain unexplored. Therefore, the aims of this study were: 1) to investigate associations between grey matter (GM) loss across the AD continuum and the distribution of multiple neurotransmitter receptors/transporters; 2) to investigate the impact of polygenic risk scores for AD (PRSs) on such associations.

**Method:**

T1‐weighted MRI scans, genetic and clinical data were selected for 800 ADNI participants (age = 74.1±7.6; M = 55.3%) including 203 cognitively unimpaired older adults (CU), 442 with mild cognitive impairment (MCI) and 155 with AD. GM atrophy was investigated in MCI and AD groups, compared with CU. JuSpace was used to calculate correlations between GM atrophy and the distribution of several neurotransmitters. Two PRSs, with (AD‐PRS) and without APOE (AD‐PRS_noAPOE_) were calculated using a Bayesian approach and investigated as predictors of the strength of correlation between GM volume (GMV) and neurotransmitters in general linear models.

**Result:**

GM atrophy was primarily in medio‐temporal areas in MCI, while was widespread in AD participants. In both groups, atrophy was negatively associated with serotoninergic and dopaminergic receptors/transporters. In the whole sample, AD‐PRS and AD‐PRS_noAPOE_ were negatively associated with the strength of correlation between GMV and 2 serotonin receptors (5‐HT1a and 5‐HT4). AD‐PRS was also associated with GMV‐FDOPA (negatively) and with GMV‐5‐HT1b (positively) correlation coefficients. In amyloid positive participants, AD‐PRS was associated with GMV‐5‐HT1b (b = 0.012, p < 0.001) and AD‐PRS_noAPOE_ with GMV‐5‐HT4 (b = ‐0.013, p < 0.001) correlation strength only.

**Conclusion:**

GM loss due to AD may be particularly affected by the alterations in inhibitory serotoninergic and in presynaptic dopaminergic activity, that are known to affect acetylcholine function and memory decline. Such alterations appear to be driven by polygenic risk for AD, with potential specific effects dependent on APOE genotype and amyloid status. Investigating further the impact of AD‐PRS on various neurotransmitter‐related neural alterations may help clarifying neuropathological changes in pre‐clinical AD and support early detection of people at risk of dementia.

